# The Reports of Hospitals

**Published:** 1895-02-09

**Authors:** 


### THE REPORTS OF HOSPITALS.

We have often been impressed with the feeling that it would be useful for the more active-minded secretaries of hospitals and kindred institutions to procure copies of the reports of other establishments which have shown special energy during the year. In pursuance of this view we propose to indicate from time to time in THE HOSPITAL any reports where the subject has been handled in a novel way, or which contain matters of special interest. The 68th report of the Chichester Infirmary contains a table showing the percentages of the cost of ordinary expenditure, compared with the figures in "Burdett's Hospital Annual for 1894." We commend this table to the notice of secretaries generally (they will find it on page 7 of the Report) as one which might be usefully adopted in the interests of the institutions and their supporters. This report also contains advertisements, a novel departure which will enable it to be distributed free of cost. The actual sum received from advertisements is not, however, stated in the accounts. As these reports are now being made up for the year we would suggest that care should be taken to include the cost per bed per in-patient and per out-patient for the year 1894, as worked out by the hospital authorities, upon some system which should be explained. We hope the medical officers of the Chichester Infirmary will join hands with the executive and so secure a reduction of 50 per cent. in the amount now spent upon alcohol. We are sorry to notice that owing to the high rates levied by the local authorities, the expenditure under the head of rating is much higher at the Chichester Infirmary than the average at the present time. In all other respects the expenditure of this institution compares favourably with that of the provincial hospitals throughout the country.

